# Acute Stress Induces Contrasting Changes in AMPA Receptor Subunit Phosphorylation within the Prefrontal Cortex, Amygdala and Hippocampus

**DOI:** 10.1371/journal.pone.0015282

**Published:** 2010-12-08

**Authors:** Dorian Caudal, Bill P. Godsil, François Mailliet, Damien Bergerot, Thérèse M. Jay

**Affiliations:** 1 INSERM U894, Physiopathologie des Maladies Psychiatriques, Centre de Psychiatrie et Neurosciences, Paris, France; 2 Université Paris Descartes, Paris, France; Johns Hopkins University, United States of America

## Abstract

Exposure to stress causes differential neural modifications in various limbic regions, namely the prefrontal cortex, hippocampus and amygdala. We investigated whether α-amino-3-hydroxy-5-methyl-4-isoxazolepropionic acid receptor (AMPAR) phosphorylation is involved with these stress effects. Using an acute inescapable stress protocol with rats, we found opposite effects on AMPA receptor phosphorylation in the medial prefrontal cortex (mPFC) and dorsal hippocampus (DH) compared to the amygdala and ventral hippocampus (VH). After stress, the phosphorylation of Ser831-GluA1 was markedly decreased in the mPFC and DH, whereas the phosphorylation of Ser845-GluA1 was increased in the amygdala and VH. Stress also modulated the GluA2 subunit with a decrease in the phosphorylation of both Tyr876-GluA2 and Ser880-GluA2 residues in the amygdala, and an increase in the phosphorylation of Ser880-GluA2 in the mPFC. These results demonstrate that exposure to acute stress causes subunit-specific and region-specific changes in glutamatergic transmission, which likely lead to the reduced synaptic efficacy in the mPFC and DH and augmented activity in the amygdala and VH. In addition, these findings suggest that modifications of glutamate receptor phosphorylation could mediate the disruptive effects of stress on cognition. They also provide a means to reconcile the contrasting effects that stress has on synaptic plasticity in these regions. Taken together, the results provide support for a brain region-oriented approach to therapeutics.

## Introduction

Acute stress alters memory performance and executive functioning, and it also causes impairments in long-term potentiation (LTP) in the hippocampus and prefrontal cortex [1]–[5]. In contrast, exposure to acute stress facilitates LTP [6], and increases extracellular glutamate levels in the amygdala [7]. Moreover, contrasting effects of stress on plasticity have been observed when comparing the dorsal and ventral regions of the hippocampus [8]. To date, the mechanism underlying these contrasting stress effects has not been fully delineated. Recently, however, it was shown that the stress hormone corticosterone triggers increases in the surface mobility and synaptic surface content of the α-amino-3-hydroxy-5-methyl-4-isoxazolepropionic acid glutamate receptor subunit 2 (AMPAR GluA2; NC_IUPHAR, [9]) [10] during LTP in hippocampal neurons [11]. Thus, AMPARs might have an important role in the effect of acute stress on cognition and plasticity.

The phosphorylation of AMPARs is an important post-translational modification that modulates the membrane expression of these receptors, various channel properties, and synaptic plasticity [12]. During LTP, site-specific phosphorylation of AMPARs prompts their recruitment to the postsynaptic membrane, which produces an increase in synaptic AMPAR function [13], [14]. Here we investigated the impact of acute stress on the phosphorylation state of different AMPAR subunits in the mPFC, hippocampus, and amygdala. Specifically, we examined the phosphorylation of GluA1 at the CaMKII and PKA sites (Ser831-GluA1 and Ser845-GluA1) and the phosphorylation of GluA2 at the tyrosine kinase and PKC sites (Tyr876-GluA2 and Ser880-GluA2) after acute platform stress. We observed opposite effects of stress on GluA1 phosphorylation in the mPFC and DH when compared to the amygdala and VH. Also, the Tyr876-GluA2 and Ser880-GluA2 sites were observed to be less phosphorylated in amygdala, whereas in the mPFC, the Ser880-GluA2 site was more phosphorylated compared to controls.

## Materials and Methods

### Animals

Experiments were performed with adult male Sprague-Dawley rats (275–300 g) that were housed four per cage. Rats were maintained on a 12/12 h light/dark schedule (lights on at 7:00 am) in a temperature controlled facility (22°C±1°C) with free access to food and water. The experiments were performed during the light phase (between 9:00 and 12:00 am) at least one week after arrival from the supplier (*Charles River*, *L'Arbresle*, *France*). All procedures were conducted in conformity with National (JO 887–848) and European (86/609/EEC) rules for animal experimentation.

### Stress protocol

The behavioral stress protocol has been described elsewhere (Rocher et al., 2004). Briefly, rats were placed on an elevated and unsteady platform for 30 min. The platform was positioned 1 m above the ground and illuminated with a high intensity light source (1500 Lux). While on the platform, animals showed urination, defecation, grooming and freezing. Immediately after the stress procedure, rats were anaesthetized with sodium pentobarbital (60 mg/kg i.p.) and then returned to their home cage until being sacrificed. Control rats (non-stressed rats) were injected while being held inside their home cage. Thirty minutes after the end of stress session, rats were killed by decapitation. Body temperature was maintained at 37°C by a homoeothermic warming blanket during this period.

### Corticosterone immunoassay

The plasma level of corticosterone was assessed as a biomarker of stress in all experiments. Blood samples were collected from the orbital sinus 10 min after anesthesia in control or stressed rats. Samples were centrifuged at 1000× g for 15 min, and serum stored at −20°C. Plasma corticosterone was assessed by immunoassay (*Corticosterone Immunoassay®*, *DSL*, *Webster*, *Texas*, *USA*).

### Brain sample preparation

After decapitation, brains were snap-frozen in liquid nitrogen as previously described [15] and stored at −80°C until processed. Using hole punchers of either 0.75 mm or 1 mm diameter (*Harris Unicore*, *Redding*, *California*, *USA*), samples from different brain regions were extracted from 100 micrometer thick brain sections in a cryostat (CM3050S, *Leica*, *Nanterre*, *France*) at −20°C. The mPFC was sampled from Bregma 4 mm to 2.7 mm, the amygdala from Bregma −2 mm to −3.5 mm, the DH from Bregma −3. mm to −4.6 mm and the VH from Bregma −5 mm to −6 mm. Samples were immediately sonicated in 1% sodium dodecyl sulfate (SDS), 10 mM NaF and 1 mM Na_3_VO_4_, and boiled for 10 min. Protein concentrations were determined with a BCA kit using a Nanodrop ND-1000 spectrophotometer (*Thermo Scientific*, *Illkirch*, *France*). Each sample was resuspended in homogenisation buffer (Tris-base 250 mM, glycerol 40%, SDS 8%, β-mercaptoethanol 20%, bromophenol blue 0.1%). In order to confirm anatomical localization, brain tissue sections that were neighbouring the brain punch sections were fixed, mounted on slides, and stained with cresyl violet.

### Western blotting

Twenty five micrograms of each sample were separated by SDS-PAGE using a 10% running gel (*Criterion™ Precast Gel*, *10% Tris-HCl*, *Bio-Rad*, *Marnes-la-Coquette*, *France*) and transferred to a 0.2 µm PVDF membrane (*Bio-Rad*, *Marnes-la-Coquette*, *France*). The membranes were then incubated for 30 min at room temperature in blocking buffer (TBS-Tween 20 0.1%, BSA 5%, NaN_3_ 0.02%). Immunoblotting was carried out overnight at 4°C with phosphorylation-state-specific antibodies against Ser831-GluA1 (*Millipore*, *Molsheim*, *France*), Ser845-GluA1 (*Millipore*, *Molsheim*, *France*), Tyr876-GluA2 (*Cell Signaling Technology Inc.*, *Danvers*, *Massachusetts*, *USA*), Ser880-GluA2 (*Interchim*, *Montluçon*, *France*). Immunoblotting was also carried out on the same stripped membranes with antibodies that were not phosphorylation-state-specific against total GluA1, GluA2 (*Millipore*, *Molsheim*, *France*) in blocking buffer. Membranes were washed three times with TBS-Tween 20 0.1% and incubated with secondary HRP anti-rabbit antibody for 1 h at room temperature. At the end of the incubation, membranes were washed three times with TBS-Tween 20 and the immunoreactive bands were detected by chemiluminescence (Immun-Star™ WesternC™ kit, *Bio-Rad*, *Marnes-la-Coquette*, *France*). A series of primary, secondary antibody dilutions and exposure times were used to optimize the experimental conditions for the linear sensitivity range of the autoradiography films (*Santa Cruz Biotechnology*, *Santa Cruz*, *California*, *USA*). Films were scanned on the GS-800 Imaging Densitometer (*Bio-Rad*, *Marnes-la-Coquette*, *France*) and the density of each band was quantified using the Quantity One software (*Bio-Rad*, *Marnes-la-Coquette*, *France*). Levels of the protein β-actin were used as an internal standard in a pilot experiment in our laboratory. Using a subset of samples, we determined that stress did not alter total levels of GluA subunits (data not shown).

### Data analysis

For the analysis of the western blotting data, the levels of GluA phosphorylated subunits were normalized to total GluA levels, based on a previous method [16]. Phosphorylation data were analyzed with two-tailed unpaired Student's T-test to evaluate statistical differences. Corticosterone measurements data were analyzed using a Mann-Whitney U test because their distribution called for a non-parametric analysis. A p-value less than 0.05 was considered significant (*). Data are expressed as the mean ± standard error of mean (SEM).

## Results

### Acute stress induces an increase in plasma corticosterone concentration

Rats placed on the elevated platform showed a significant and dramatic increase in plasma corticosterone levels when compared to non-stressed rats (*n* = 12; 830.3±112.7 ng/mL and 161.5±45.3 ng/mL in stressed and non-stressed rats, respectively; U = 3, *p*<0.001). The sensitivity of the corticosterone assay was 1.6 ng/mL.

### Acute stress exposure decreases phosphorylation of GluA1 in the mPFC and DH

**Figure 1:**
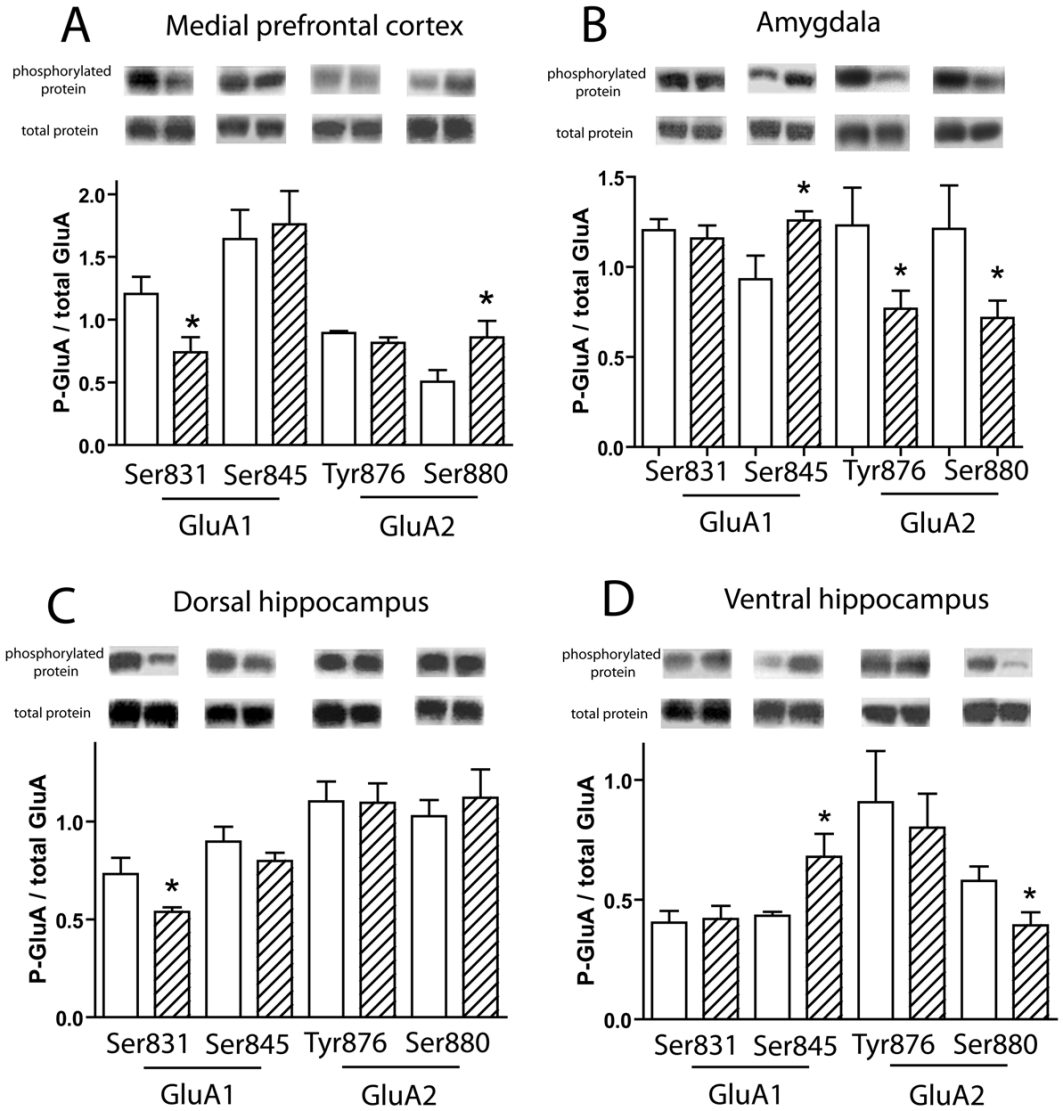
Phosphorylation states at multiple AMPAR subunits after acute stress. Immunoblots and histograms of the ratio between the phosphorylated form and total amount of the protein (white bars: no stress group, cross hatched bars: stress group). In the mPFC (A), stress decreased Ser831-GluA1 phosphorylation (p<0.05) and increased Ser880-GluA2 phosphorylation (p<0.05). In the amygdala (B), stress increased Ser845-GluA1 phosphorylation (p<0.05) and decreased Tyr876-GluA2 and Ser880-GluA2 phosphorylation (p<0.05. In the DH (C), stress decreased Ser831-GluA1 phosphorylation (p<0.05). In the VH (D), stress increased Ser845-GluA1 phosphorylation (p<0.05) and decreased Ser880-GluA2 phosphorylation (p<0.05). Immunoblots (100 kDa) illustrate the phosphorylated form of the protein (left: control, right: stress). The number of animals per group is 10 for controls and 11 for stress. Unpaired, two-tailed, Student's T-test. *, p<0.05, compared with the corresponding control group.

AMPAR GluA1 and GluA2 subunits phosphorylation states were studied in the mPFC, including both prelimbic and infralimbic areas, and in the DH. Thirty minutes after the end of stress, a significant decrease in the phosphorylation level of Ser831-GluA1 was found in mPFC and DH of stressed animals compared to controls (t (19) = 2.438, p<0.05 and t (19) = 2.347, p<0.05, respectively; Fig. 1A and 1C), whereas no significant changes were observed for Ser845-GluA1 and Tyr876-GluA2 phosphorylation states (Fig. 1A and 1C). In contrast, acute stress caused an increase in Ser880-GluA2 phosphorylation in the mPFC (t (19) = 2.196, p<0.05; Fig. 1A), but not in the DH (Fig. 1C).

### Acute stress exposure increases the phosphorylation of GluA1 and decreases the phosphorylation of GluA2 in the amygdala and VH

AMPAR GluA1 and GluA2 subunit phosphorylation states were studied in the amygdala and VH. Thirty minutes after the end of stress, changes in the phosphorylation sites of both GluA1 and GluA2 were found, with a decreased phosphorylation level at the Tyr876-GluA2 and Ser880-GluA2 sites in the amygdala (t (23) = 2.207, p<0.05 and t (23) = 2.167, p<0.05, respectively; Fig. 1B). A decreased phosphorylation level of Ser880-GluA2 site in the VH was found, (t (19) = 2.174, p<0.05; Fig. 1D), as opposed to the increased phosphorylation level of Ser880-GluA2 site observed in the mPFC (t (19) = 2.196, p<0.05; Fig. 1A). In contrast to what was observed in the mPFC for Ser831-GluA1, stress increased the phosphorylation states of Ser845-GluA1 in the amygdala and VH (t (19) = 2.891, p<0.05; Fig. 1B and 1D). All the phosphorylation changes on GluA1 and GluA2 subunits after stress are summarized in Table 1.

**Table 1:** Summary of changes in AMPAR phosphorylation of GluA1 and GluA2 subunits after stress in the four regions studied.

	GluA1		GluA2	
	Ser 831(CamKII/PKC)	Ser 845(PKA)	Tyr 876(Tyr kinase)	Ser 880(PKC)
Medial prefrontal cortex	**↓** (38.6%)	**↔**	**↔**	**↑** (70.1%)
Amygdala	**↔**	**↑** (32.8%)	**↓** (37.5%)	**↓** (40.9%)
Dorsal hippocampus	**↓** (26.5%)	**↔**	**↔**	**↔**
Ventral hippocampus	**↔**	**↑** (57.2%)	**↔**	**↓** (32.2%)

## Discussion

Here we report that exposure to acute stress causes a unique profile of AMPAR phosphorylation in the mPFC, amygdala and hippocampus. Decreases in the phosphorylation of the CaMKII/PKC Ser831-GluA1 site were observed in the mPFC and DH, whereas increases in Ser845-GluA1 phosphorylation were seen in the amygdala and VH. Acute stress also modulated the phosphorylation state of the GluA2 subunit with phosphorylation decreases at both the Tyr876-GluA2 and Ser880-GluA2 sites in the amygdala, but only Ser880-GluA2 in the VH, as well as an increase in the phosphorylation of Ser880-GluA2 in the mPFC. Thus, acute stress differentially modulates AMPAR phosphorylation in several parts of the corticolimbic system.

Previous work from many labs indicates that GluA1 AMPAR subunit phosphorylation at the Ser831-GluA1 and Ser845-GluA1 sites can mediate the generation of LTP [14]. The phosphorylation on Ser831-GluA1 is thought to affect plasticity by increasing the AMPAR channel conductance [17] and potentiating GluA1-mediated current [18], [19], whereas the phosphorylation of Ser845-GluA1 accompanies surface insertion of AMPARs receptors at in extra synaptic sites, which provides a pool of receptors available for LTP induction [20], [21]. Furthermore, other research has demonstrated the acute stress prevents the induction of LTP in DH and mPFC [4], [22]–[24]. In light of these previous findings, our present results suggest that the mechanism by which acute stress disrupts LTP in these regions involves the reduction of Ser831-GluA1 phosphorylation. Using similar reasoning, the increase in Ser845-GluA1 phosphorylation observed within the amygdala might explain how acute stress enhances LTP in that region [6]. Thus, our results provide a framework for explaining the contrasting effects that stress has on LTP in different brain regions. Namely, these effects occur because acute stress differentially modulates GluA1 AMPAR subunit phosphorylation, with decreased phosphorylation seen in the DH and mPFC and increased phosphorylation seen in the amygdala. Moreover, the contrasting results we obtained for the GluA1 phosphorylation in the DH and the VH, which were manifested as a decrease of Ser831-GluA1 phosphorylation and an increase of Ser845-GluA1 phosphorylation, respectively, provide support for the recent hypothesis that stress differentially regulates ventral and dorsal routing within the hippocampus [8], [25]. Such a functional segmentation, with the VH being involved with emotion and the DH regulating information processing, is supported by evidence from numerous anatomical, behavioral and physiological studies [26], [27].

In addition to affecting GluA1 subunits, we observed that acute stress modulates the phosphorylation state of the GluA2 subunit in amygdala, mPFC and VH. Exposure to stress decreased phosphorylation at both the Tyr876-GluA2 and Ser880-GluA2 sites in the amygdala, and at the Ser880-GluA2 site in the VH. Phosphorylation of the Tyr876-GluA2 residue is known to control the surface expression and the synaptic targeting of the GluA2 subunit by causing its internalization [28], whereas Ser880-GluA2 phosphorylation decreases the affinity of GluA2 for GRIP and then triggers its internalization [29]. Each of these effects is thought to contribute to the reduced synaptic strength observed in long-term depression [30]–[32]. Thus, acute stress likely reduces GluA2 AMPAR internalization in the amygdala and in the VH, which predicts that acute stress would impede the generation of LTD in these regions. In contrast, the increased phosphorylation of the Ser880-GluA2 residue in the mPFC that we observed suggests acute stress increases AMPAR internalization and facilitates the generation of LTD in that region.

Previous work has shown that corticosterone facilitates the recruitment of GluA2-AMPARs by enhancing lateral diffusion, and increases both GluA2-AMPAR synaptic surface content and surface mobility in cultured hippocampal neurons [10], [11]. A recent report has also demonstrated that a short treatment of corticosterone increases AMPAR-mediated synaptic transmission and AMPAR trafficking in cultured PFC neurons [33]. In contrast, while the elevated platform stress protocol causes a dramatic increase in plasma corticosterone levels, we observed a decrease in phosphorylation of AMPA receptors in the PFC, which would be expected to have the opposite effect on glutamatergic processes. Additionally, we have observed that the blockade of glucocorticosteroid receptors protects against the stress-induced disruption of LTP in the mPFC synapses after stress [34]. It may be that a massive influx of corticosterone at PFC neurons perturbs that conditions in which the induction of LTP is possible. This hypothesis is consistent with the finding that exposure to stress can prevent the induction of LTP and favour the induction of long-term depression [24]. Future studies are needed to clarify the influence of glucocorticoids on site-specific phosphorylation of AMPARs in the different brain regions.

Our laboratory has also previously demonstrated that post-stress treatment with the antidepressant drug tianeptine protects against the stress-induced disruption of neural plasticity [4]. Recent work with phosphomutant GluA1 mice, which have point mutations at both the Ser831-GluA1 and Ser845-GluA1 sites, suggests that this antidepressant-like drug effect might be related to AMPAR GluA1 subunit phosphorylation. Specifically, fluoxetine administration was seen to increase GluA1 phosphorylation at the Ser845-GluA1 site, whereas tianeptine treatment increased GluA1 phosphorylation at the Ser831-GluA1 site in the frontal cortex and hippocampus, as well as at the Ser845-GluA1 site in the hippocampus [16], [35]. Therefore, tianeptine's ability to protect LTP from stress-induced disruption might be related to its capacity to augment AMPAR GluA1 phosphorylation [16], [35]. Given the robust clinical relationship between stress and major depression, the present data further emphasize a potential beneficial role of AMPA receptor modulators in the treatment of mood disorders.

Our results might also have implications for the impact of stress on learning and memory. As has been discussed, various stressors modulate the induction of synaptic plasticity in the hippocampus and PFC [23], [24], [36], [37], and tianeptine treatment increases phosphorylation at the Ser831 site of the GluA1 receptor subunit [16], [35]. It is also true that exposure to stress disrupts working memory [1], [38], and knock-out mice that lack the GluA1 subunit show dramatic impairments in spatial working memory [39]. These results support the hypothesis that the phosphorylation state of GluA1 receptor subunits at the Ser831 site is a key characteristic of the stress-induced dysregulation in synaptic plasticity that may lead to the disruption of working memory. Classically, the PFC has been treated as a key brain region for working memory [40], [41], yet this view has been challenged more recently with evidence supporting the hippocampus' predominant role [39]. It might be that GluA1 subunits within the VH-to-mPFC pathway have an important role for the integration of stress effects on working memory and on plasticity.

In a recent paper, Fumagalli and colleagues provided evidence that GluA1 subunits are necessary for stress to cause an increase in the phosphorylation of the NMDARs [42]. The classical mechanism that is thought to govern the interaction between NMDARs and AMPARs during synaptic plasticity, however, involves a sequence where the activation of NMDARs leads to the modification of AMPARs [43]–[46]. Here we have reported that stress induces a decrease in Ser831-GluA1 phosphorylation. Previous work from hippocampal slice physiology indicates that NMDAR blockade can cause a similar decrease in Ser831-GluA1 phosphorylation [47]. Based on this observation we hypothesize that stress may have a direct effect on NMDARs, which then leads to downstream modification of AMPARs phosphorylation. In contrast, Fumagalli et al. reported that exposure to acute restraint stress increased the phosphorylation of both the GluN1 and GluN2B subunits of NMDARs in the hippocampus of wild-type mice, but not in mutant mice [42]. This finding implies that AMPARs may have an unappreciated role in governing NMDARs during stress [48]. It might also be that the absence of AMPAR GluA1 subunit resulted in a compensatory regulation mechanism for NMDARs because AMPARs and NMDARs are tightly coregulated by activity at the synapses.

We have previously reported that elevated platform stress does not lead to a general modulation of GluA1 phosphorylation in brain tissue sampled from the entire frontal cortices [35]. The present methodology targeted a more-specific brain region and we observed that exposure to elevated platform stress decreased the phosphorylation of Ser831 on the GluA1 subunit in the mPFC. Together, these finding support our hypothesis that exposure to elevated platform stress leads to a regionally-specific profile of phosphorylation changes. Yet, in contrast to our results, Fumagalli and colleagues reported a stress-induced increase in GluA1 phosphorylation at the Ser831 site in the hippocampus of mice after restraint stress [42] and a similar pattern has been reported in the hippocampus of rats after acute swim stress [49]. These findings highlight the complexity of the brain's response to stress, and they imply that modulations on AMPAR phosphorylation may depend on the species, the stress procedure, and the brain region studied.

In conclusion, the present findings provide evidence that exposure to acute stress induces changes in AMPAR functioning that are subunit-specific and brain region-specific. These findings provide a framework for explaining the pattern that acute stress disrupts the generation of LTP in mPFC and DH, while it simultaneously facilities LTP in the amygdala. Given that stress plays a significant role in the pathophysiology of affective disorders, it seems likely that successful pharmacological treatments will need to deal with these subunit-specific and region-specific changes in glutamatergic actions that occur after stress. Thus, from a therapeutic point of view, our results highlight the need for a region-oriented approach. Our results also imply that the dysregulation of glutamatergic activity in the limbic-cortical system is an important target for the treatment of depression [50], [51].
